# *TBK* *1*‐Associated Primary Lateral Sclerosis Followed by Right Temporal Variant Frontotemporal Dementia

**DOI:** 10.1002/acn3.70329

**Published:** 2026-02-05

**Authors:** Tomoyasu Matsubara, Naoki Kihara, Satoko Miyatake, Koji Fujita, Konoka Tachibana, Ryosuke Miyamoto, Hiroki Yamazaki, Yusuke Osaki, Nazere Keyoumu, Yuki Kuwano, Nobutoshi Morimoto, Suzuran Saito, Eriko Koshimizu, Yoichi Otomi, Kenji Ishibashi, Masafumi Harada, Naomichi Matsumoto, Hiroyuki Morino, Yuishin Izumi

**Affiliations:** ^1^ Department of Neurology Tokushima University Hospital Tokushima Japan; ^2^ Department of Clinical Neuroscience and Therapeutics, Graduate School of Biomedical and Health Sciences Hiroshima University Hiroshima Japan; ^3^ Department of Clinical Genetics Yokohama City University Hospital Yokohama Japan; ^4^ Department of Human Genetics Yokohama City University Graduate School of Medicine Yokohama Japan; ^5^ Department of Neurogenetics, Molecular Neuroscience Research Center Shiga University of Medical Science Otsu Shiga Japan; ^6^ Department of Neurology Tokushima University Graduate School of Biomedical Sciences Tokushima Japan; ^7^ Clinical Research Center for Developmental Therapeutics Tokushima University Hospital Tokushima Japan; ^8^ Department of Medical Genetics Tokushima University Graduate School of Biomedical Sciences Tokushima Japan; ^9^ Department of Neurology Kagawa Prefectural Central Hospital Takamatsu Japan; ^10^ Department of Radiology Tokushima University Graduate School of Biomedical Sciences Tokushima Japan; ^11^ Diagnostic Neuroimaging Research, Research Team for Neuroimaging Tokyo Metropolitan Institute for Geriatrics and Gerontology Tokyo Japan; ^12^ Department of Rare Disease Genomics Yokohama City University Hospital Yokohama Japan; ^13^ Medical Genome Center National Center of Neurology and Psychiatry Kodaira, Tokyo Japan

**Keywords:** frontotemporal dementia, motor neuron disease, primary lateral sclerosis, *TBK1*, TDP‐43

## Abstract

We report a 58‐year‐old woman with a novel splice‐site variant in the TANK‐binding kinase 1 (*TBK1*:c.993–2A>C p.Ala332TyrfsTer39) who sequentially developed primary lateral sclerosis (PLS) followed by right temporal variant frontotemporal dementia (rtvFTD). Neuroimaging demonstrated right anterior temporal atrophy before cognitive symptoms, and prosopagnosia represented the earliest manifestation of rtvFTD. Molecular analysis revealed reduced levels of correctly spliced *TBK1* transcripts, consistent with haploinsufficiency. Given the shared involvement of TDP‐43 pathology in both PLS and rtvFTD, this case indicates *TBK1* dysfunction as a fundamental genetic factor underlying the coexistence of these phenotypes, underscoring the clinical value of early neuroimaging and genetic evaluation.

## 1 Introduction

Amyotrophic lateral sclerosis (ALS) and frontotemporal dementia (FTD) are closely related neurodegenerative disorders that may exhibit clinical overlap: 15% of patients with ALS develop FTD and 12%–14% of those with FTD develop ALS during their clinical course [1, 2, 3]. Variants in the TANK‐binding kinase 1 (*TBK1*) gene are associated with FTD and/or ALS (FTDALS4; OMIM #616439), making *TBK1* a major genetic contributor to the FTD–ALS spectrum alongside *C9orf72*. Pathogenic variants in *TBK1* commonly present as behavioral variant FTD (bvFTD), primary progressive aphasia, ALS, or combinations of these syndromes [4, 5, 6].

Beyond these common presentations, *TBK1* is associated with a broader phenotypic spectrum, including primary lateral sclerosis (PLS), an upper motor neuron‐predominant condition within the ALS spectrum, and right temporal variant FTD (rtvFTD), a socioemotional‐predominant condition within the FTD spectrum characterized by predominant right anterior temporal degeneration. While these observations suggest substantial phenotypic diversity among *TBK1* variant carriers, only a limited number of such cases have been reported [7, 8, 9, 10, 11, 12, 13] and the full clinical spectrum has yet to be delineated. In this context, we describe a patient harboring a novel pathogenic splice‐site variant in *TBK1* who sequentially developed PLS and rtvFTD.

## 2 Case Description

**FIGURE 1:**
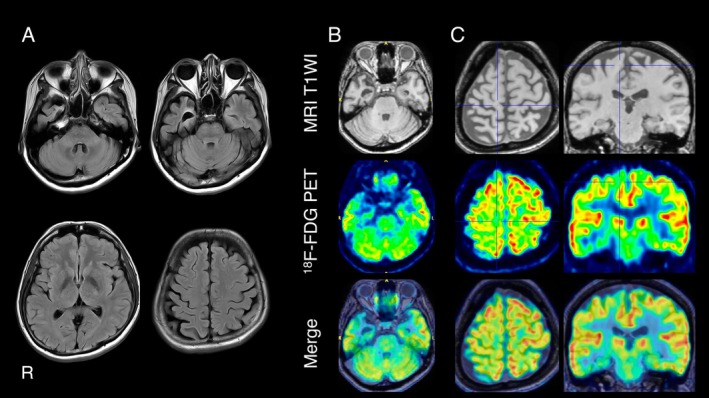
Magnetic resonance imaging and [^18^F]‐2‐fluoro‐2‐deoxy‐D‐glucose positron emission tomography (^18^F‐FDG PET) imaging. (A) Axial fluid attenuated inversion recovery (FLAIR) sequence magnetic resonance images obtained when the patient was at age 57. Atrophy is observed in the anterior and medial regions of the right temporal lobe. (B) ^18^F‐FDG PET brain images show hypometabolism predominantly in the anterior and medial regions of the right temporal lobe. (C) The medial region of the precentral gyrus (indicated by the blue cross mark) shows right‐predominant hypometabolism. The laterality and medial predominance of the metabolic reduction in the precentral gyrus were consistent with the patient's clinical manifestations.

A 57‐year‐old right‐handed woman presented with gait disturbance that began at age 56. She had no significant past medical history and no family history of neurological diseases. Neurological examination revealed marked spasticity in the lower limbs. Deep tendon reflexes were brisk in all four limbs, more pronounced on the left, and bilateral extensor plantar responses were elicited. Muscle weakness was observed in the hamstrings and tibialis anterior muscles, predominantly on the left, without muscle atrophy or fasciculation. Cranial nerves were intact, and the jaw reflex was normal. Extrapyramidal signs, ataxia, sensory deficits, and autonomic dysfunction were absent. At the initial visit, the patient showed no evidence of cognitive impairment, with scores as follows: Mini‐Mental State Examination (MMSE) 30/30, Montreal Cognitive Assessment (MoCA) 29/30, Frontal Assessment Battery (FAB) 17/18, Addenbrooke's Cognitive Examination‐Revised (ACE‐R) 95/100, and Western Aphasia Battery Aphasia Quotient (WAB‐AQ) 97/100. Blood tests were unremarkable except for elevated serum creatine kinase (426 IU/mL). Cerebrospinal fluid analyses, including Aβ_1–42_, phosphorylated tau 181, and total tau, showed no abnormalities. Nerve conduction studies, needle electromyography (EMG), and neuromuscular ultrasound revealed no abnormalities. Spinal magnetic resonance imaging (MRI) was unremarkable, while brain MRI revealed atrophy in the right anterior temporal lobe (Figure 1A).

Six months after the initial visit, the patient developed prosopagnosia as confirmed by the Visual Perception Test for Agnosia and Famous Faces Test version 2 (VPTA‐FFT ver. 2). Lower limb spastic paresis worsened progressively, necessitating the use of a walker. Within 1 year of the initial visit, she also developed grip difficulty. However, follow‐up EMG and ultrasound show no evidence of lower motor neuron involvement. Persistence of upper motor neuron involvement without lower motor neuron involvement for over 2 years supported a diagnosis of probable PLS based on the consensus diagnostic criteria [14]. She also developed a depressive affect accompanied by delusions of guilt.

By age 58, the patient developed mild cognitive decline (MMSE 27/30 [recall 0/3], MoCA 24/30 [recall 0/5], and FAB 12/18) and met the criteria for rtvFTD [15]; she was diagnosed at a very early stage of the disease. In further support of this diagnosis, [^18^F]‐2‐fluoro‐2‐deoxy‐D‐glucose positron emission tomography (^18^F‐FDG PET) demonstrated severe hypometabolism in the right anterior temporal lobe. It also revealed hypometabolism in the medial region of the precentral gyrus, with right‐side predominance, consistent with her clinical manifestations (Figure 1B,C).

**FIGURE 2:**
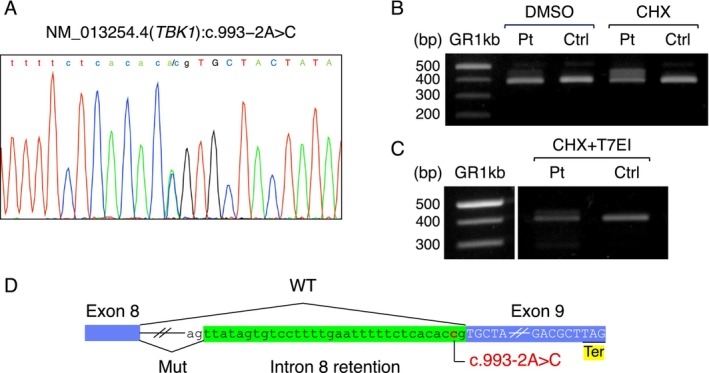
Genetic findings: *TBK1* c.993–2A>C produces intron 8 retention and frameshift. (A) Sanger chromatogram of a genomic DNA sequence showing the heterozygous A to C substitution at position c.993–2 in the patient. (B) Reverse transcription polymerase chain reaction (RT‐PCR) of lymphoblastoid‐cell RNA reveals wild‐type and intron 8‐retained bands in the patient but not in the control; cycloheximide treatment of the cells enhances the aberrant band, indicating the involvement of partial nonsense‐mediated decay. Note that RT‐PCR amplicons from cycloheximide‐treated lymphoblastoid cell lines show wild‐type, intron‐retained, and heteroduplex bands. (C) RT‐PCR amplicons from cycloheximide‐treated lymphoblastoid cell lines are further treated with T7 endonuclease I to remove heteroduplexes and improve clarity. (D) Schematic illustration of the loss of the canonical exon 9 acceptor (ag), intron 8 retention (green), and a premature stop codon (yellow) predicting p.Ala332TyrfsTer39. bp, base pairs; CHX, cycloheximide; Ctrl, control; DMSO, Dimethyl sulfoxide; Mut, mutant; Pt, patient; T7EI, T7 endonuclease I; Ter, premature termination codon; WT, wild type.

Genetic testing was performed because of clinical suspicion of an FTD–ALS spectrum. A detailed description of the analysis methods is provided in the Supporting Information. A novel heterozygous splice‐site variant in *TBK1* (NM_013254.4:c.993–2A>C) was identified and confirmed by Sanger sequencing (Figure 2A). *In silico* prediction indicated that the variant would be deleterious, with a Combined Annotation Dependent Depletion (CADD) Phred score [16] of 34. According to the American College of Medical Genetics and Genomics (ACMG)/Association for Molecular Pathology (AMP) criteria [17], the variant was classified as pathogenic (PVS1, PM2, PP3).

Reverse transcription polymerase chain reaction (RT‐PCR) of patient‐derived lymphoblastoid cell lines revealed a reduction in the correctly spliced transcript compared with the control. A faint, slightly larger amplicon retaining intron 8 was also detected, indicating an aberrant transcript (p.Ala332TyrfsTer39). The abundance of the aberrant transcript increased after cycloheximide treatment, suggesting partial nonsense‐mediated mRNA decay (Figure 2B–D). These findings support a loss‐of‐function effect consistent with TBK1 haploinsufficiency, strengthening the causal link between TBK1 dysfunction and the observed disease phenotype. No other pathogenic variants or *C9orf72* hexanucleotide repeat expansion were detected.

## 3 Discussion

This study describes a patient with sequential development of PLS followed by rtvFTD, harboring a novel pathogenic splice‐site variant in *TBK1* (c.993–2A>C p.Ala332TyrfsTer39). While *TBK1* variants are well‐established causes of ALS and bvFTD [4, 5], their association with PLS or rtvFTD has rarely been reported. To date, 7 cases of PLS associated with *TBK1* variants have been documented, of which 6 were described as PLS without FTD (Table S1) [7, 8, 9, 10, 11]. The remaining case exhibited the coexistence of PLS and prosopagnosia; however, detailed clinical information, including neuroimaging findings, was unavailable [10]. Right temporal lobe atrophy associated with *TBK1* variants has been reported in 5 cases, all of which exhibited a bvFTD‐like phenotype, whereas none were associated with PLS (Table S2) [12, 13]. In contrast to previous reports, the present case uniquely demonstrates the subsequent development of rtvFTD after PLS onset, providing longitudinal evidence that these phenotypes can coexist within a single *TBK1*‐associated disease trajectory.

**FIGURE 3:**
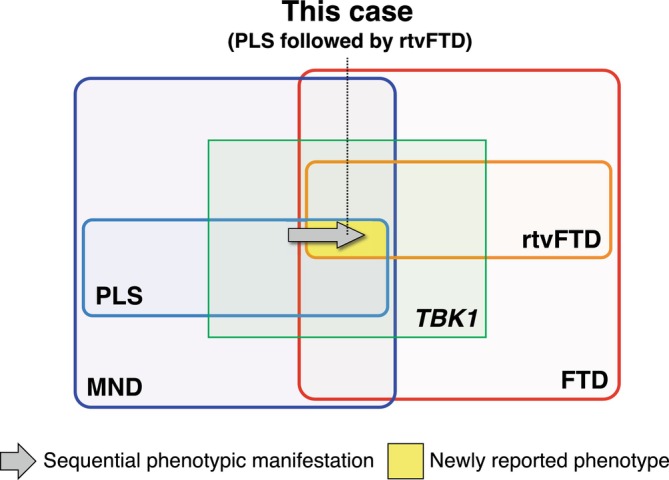
Schematic representation of the phenotypic spectrum associated with *TBK1* variants, illustrating the position of the present case. The schema shows the overlap between the motor neuron disease (MND) spectrum (blue) and the frontotemporal dementia (FTD) spectrum (red). Primary lateral sclerosis (PLS) and the right temporal variant of FTD (rtvFTD) are indicated as subgroups within these spectra. The green box represents the *TBK1*‐associated disease spectrum (*TBK1*). The yellow area denotes the newly reported phenotype in this study, where PLS was followed by rtvFTD. The gray arrow indicates the sequential phenotypic manifestation observed in this patient, with etvFTD developing in addition to PLS.

Importantly, previous reports have generally described PLS or rtvFTD as distinct phenotypes, whereas the present case demonstrates a phenotypic overlap between these two entities, supported by neuroimaging findings (Figure 3). This overlap is unlikely to be coincidental, as PLS and rtvFTD share a common disease mechanism. Both conditions are frequently characterized by TDP‐43 pathology [18, 19], and pathogenic variants in *TBK1*, which is tightly linked to TDP‐43 proteinopathy [20, 21], can bridge the motor neuron–predominant phenotype of PLS and the cognitive–behavioral phenotype of rtvFTD. Thus, TBK1 dysfunction may provide a biological link between PLS and rtvFTD phenotypes through a shared TDP‐43–mediated disease process. Comprehensive genetic analysis of *TBK1* may help elucidate the molecular basis underlying this phenotypic spectrum and refine the diagnostic framework in patients presenting with combined upper motor neuron and right anterior temporal lobe involvement.

This case provided a unique opportunity to detect early and sequential clinical manifestations of rtvFTD, as the patient was longitudinally followed from the stage of pure PLS without detectable symptoms of rtvFTD. Brain MRI obtained during this pure PLS period revealed focal atrophy in the right anterior temporal lobe. Prompted by this neuroimaging finding, proactive screening for prosopagnosia enabled its identification as the earliest clinical manifestation of rtvFTD, with memory impairment and depressive affect becoming prominent within 1 year thereafter. Prosopagnosia, memory impairment, and behavioral changes have been described as frequent presenting symptoms at the first assessment in patients with rtvFTD [15]. In the present case, prosopagnosia preceded other cognitive and behavioral symptoms, highlighting its potential value as an early clinical clue for rtvFTD. Moreover, given that behavioral abnormalities in rtvFTD typically develop approximately 3 years after onset [22], early recognition of rtvFTD guided by brain MRI findings in patients with motor neuron involvement may provide a temporal window for clinicians and caregivers to anticipate and prepare for subsequent behavioral changes.

In conclusion, we present a patient harboring a novel pathogenic splice‐site variant in *TBK1* who sequentially developed PLS followed by rtvFTD. Prosopagnosia may represent a key early feature of rtvFTD, and *TBK1* should be considered as an underlying cause in patients presenting with either PLS or rtvFTD, particularly when these conditions occur together. Early detection of the variant, informed by such clinical clues, would hold greater therapeutic relevance should gene‐specific interventions for *TBK1* become feasible in the future.

## Author Contributions

T.M. and N.K. designed and conceptualized the study, collected, analyzed, and interpreted the data, and drafted the manuscript. Y.I. designed and conceptualized the study, collected the clinical data, and revised the manuscript. K.F., H.Y., Y.O., N.M., Y.O., K.I., and M.H. collected the clinical data and revised the manuscript. S.M., K.T., R.M., N.K., Y.K., S.S., E.K., N.M., and H.M. analyzed and interpreted the genomic data and revised the manuscript. All authors read and approved the final manuscript.

## Funding

This work was supported by MHLW Research on rare and intractable diseases program Grant Number JPMH23FC1008 (YI), AMED under grant numbers JP25ek0109674, JP25ek0109760, JP25ek0109617, JP25ek0109648, JP25ek0109677 (NM), JP25dk0207066, and JP23re0122001 (YI), JSPS KAKENHI under grant numbers JP25K19028 (TM), JP23K27520 (SM) and JP24K02230 (NM), the Naito Foundation (SM), and the Takeda Science Foundation (TM, NM). The funding bodies did not play any role in the design of the study, the collection, analysis, or interpretation of the data, or the writing of the manuscript.

## Conflicts of Interest

The authors declare no conflicts of interest.

## Supporting information


**Data S1:** acn370329‐sup‐0001‐Supinfo1.docx.

**Table S1:** Literature review of primary lateral sclerosis cases harboring *TBK1* variants.

**Table S2:** Literature review of cases of right temporal lobe atrophy harboring *TBK1* variants.

## Data Availability

The data that support the findings of this study are available from the corresponding author upon reasonable request.

## References

[1] G. M. Ringholz , S. H. Appel , M. Bradshaw , N. A. Cooke , D. M. Mosnik , and P. E. Schulz , “Prevalence and Patterns of Cognitive Impairment in Sporadic ALS,” Neurology 65 (2005): 586–590. 16116120 10.1212/01.wnl.0000172911.39167.b6

[2] C. Lomen‐Hoerth , T. Anderson , and B. Miller , “The Overlap of Amyotrophic Lateral Sclerosis and Frontotemporal Dementia,” Neurology 59 (2002): 1077–1079. 12370467 10.1212/wnl.59.7.1077

[3] J. R. Burrell , M. C. Kiernan , S. Vucic , and J. R. Hodges , “Motor Neuron Dysfunction in Frontotemporal Dementia,” Brain 134 (2011): 2582–2594. 21840887 10.1093/brain/awr195

[4] I. J. Swift , M. Bocchetta , H. Benotmane , et al., “Variable Clinical Phenotype in TBK1 Mutations: Case Report of a Novel Mutation Causing Primary Progressive Aphasia and Review of the Literature,” Neurobiology of Aging 99 (2021): 100.e9–100.e15. 10.1016/j.neurobiolaging.2020.08.014 PMC7907669 32980182

[5] E. N. Aiello , S. Feroldi , G. De Luca , et al., “Primary Progressive Aphasia and Motor Neuron Disease: A Review,” Frontiers in Aging Neuroscience 14 (2022): 1003792. 36158556 10.3389/fnagi.2022.1003792 PMC9492890

[6] G. Tohnai , R. Nakamura , J. Sone , et al., “Frequency and Characteristics of the TBK1 Gene Variants in Japanese Patients With Sporadic Amyotrophic Lateral Sclerosis,” Neurobiology of Aging 64 (2018): 158.e15–158.e19. 10.1016/j.neurobiolaging.2017.12.005 29398122

[7] E. Gómez‐Tortosa , J. Van der Zee , M. Ruggiero , et al., “Familial Primary Lateral Sclerosis or Dementia Associated With Arg573Gly TBK1 Mutation,” Journal of Neurology, Neurosurgery, and Psychiatry 88 (2017): 996–997. 28365590 10.1136/jnnp-2016-315250

[8] M. R. Costa , M. Gromicho , A. C. Pronto‐Laborinho , G. Miltenberger Miltényi , and M. de Carvalho , “Novel TBK1 LoF Variant in a Family With Upper Motor Neuron Predominant Motor Neuron Disease,” Journal of the Neurological Sciences 403 (2019): 117–118. 31276859 10.1016/j.jns.2019.06.029

[9] P. Corcia , C. Lunetta , P. Couratier , et al., “Familial Clustering of Primary Lateral Sclerosis and Amyotrophic Lateral Sclerosis: Supplementary Evidence for a Continuum,” European Journal of Neurology 28 (2021): 2780–2783. 34110677 10.1111/ene.14960

[10] E. M. J. de Boer , B. S. de Vries , M. Pennings , et al., “Genetic Characterization of Primary Lateral Sclerosis,” Journal of Neurology 270 (2023): 3970–3980. 37133535 10.1007/s00415-023-11746-7 PMC10345048

[11] A. Manini , A. Brusati , M. Grassano , et al., “Whole Genome Sequencing Analysis in Primary Lateral Sclerosis (PLS) Patients Reveals Mutations in Neurological Diseases‐Causing Genes,” Journal of Neurology 272 (2025): 587. 40844737 10.1007/s00415-025-13328-1

[12] S. Van Mossevelde , J. van der Zee , I. Gijselinck , et al., “Clinical Features of TBK1 Carriers Compared With C9orf72, GRN and Non‐Mutation Carriers in a Belgian Cohort,” Brain 139 (2016): 452–467. 26674655 10.1093/brain/awv358 PMC4805085

[13] C. A. Koriath , M. Bocchetta , E. Brotherhood , et al., “The Clinical, Neuroanatomical, and Neuropathologic Phenotype of TBK1‐Associated Frontotemporal Dementia: A Longitudinal Case Report,” Alzheimers Dement (Amst) 6 (2017): 75–81. 28229125 10.1016/j.dadm.2016.10.003 PMC5312484

[14] M. R. Turner , R. J. Barohn , P. Corcia , et al., “Primary Lateral Sclerosis: Consensus Diagnostic Criteria,” Journal of Neurology, Neurosurgery, and Psychiatry 91 (2020): 373–377. 32029539 10.1136/jnnp-2019-322541 PMC7147236

[15] H. Ulugut Erkoyun , C. Groot , R. Heilbron , et al., “A Clinical‐Radiological Framework of the Right Temporal Variant of Frontotemporal Dementia,” Brain 143 (2020): 2831–2843. 32830218 10.1093/brain/awaa225 PMC9172625

[16] M. Schubach , T. Maass , L. Nazaretyan , S. Röner , and M. Kircher , “CADD v1.7: Using Protein Language Models, Regulatory CNNs and Other Nucleotide‐Level Scores to Improve Genome‐Wide Variant Predictions,” Nucleic Acids Research 52 (2024): D1143–D1154. 38183205 10.1093/nar/gkad989 PMC10767851

[17] S. Richards , N. Aziz , S. Bale , et al., “Standards and Guidelines for the Interpretation of Sequence Variants: A Joint Consensus Recommendation of the American College of Medical Genetics and Genomics and the Association for Molecular Pathology,” Genetics in Medicine 17 (2015): 405–424. 25741868 10.1038/gim.2015.30 PMC4544753

[18] I. R. A. Mackenzie and H. Briemberg , “TDP‐43 Pathology in Primary Lateral Sclerosis,” Amyotroph Lateral Scler Frontotemporal Degener 21 (2020): 52–58. 32657153 10.1080/21678421.2020.1790607

[19] H. Ulugut , A. A. Dijkstra , M. Scarioni , et al., “Right Temporal Variant Frontotemporal Dementia Is Pathologically Heterogeneous: A Case‐Series and a Systematic Review,” Acta Neuropathologica Communications 9 (2021): 131. 34344452 10.1186/s40478-021-01229-z PMC8330072

[20] W. Shao , T. W. Todd , Y. Wu , et al., “Two FTD‐ALS Genes Converge on the Endosomal Pathway to Induce TDP‐43 Pathology and Degeneration,” Science 378 (2022): 94–99. 36201573 10.1126/science.abq7860 PMC9942492

[21] E. M. J. de Boer , V. K. Orie , T. Williams , et al., “TDP‐43 Proteinopathies: A New Wave of Neurodegenerative Diseases,” Journal of Neurology, Neurosurgery, and Psychiatry 92 (2020): 86–95. 33177049 10.1136/jnnp-2020-322983 PMC7803890

[22] W. W. Seeley , A. M. Bauer , B. L. Miller , et al., “The Natural History of Temporal Variant Frontotemporal Dementia,” Neurology 64 (2005): 1384–1390. 15851728 10.1212/01.WNL.0000158425.46019.5C PMC2376750

