# Supplementary material for: TBK 1‐Associated Primary Lateral Sclerosis Followed by Right Temporal Variant Frontotemporal Dementia
Source: Ann Clin Transl Neurol. 2026 Feb 5;13(5):1073–7. doi: 10.1002/acn3.70329 (PMC13161887; doi:10.1002/acn3.70329)
Supplement: Supplementary file 1 — Data S1: acn370329‐sup‐0001‐Supinfo1.docx. [file ACN3-13-1073-s002.docx]

**Supplementary Information**

**Genetic analysis**

Genomic DNA was extracted from the patient blood specimen. For whole-exome sequence analysis, genomic DNA was captured using the Twist Exome 2.0 kit (Twist Bioscience, South San Francisco, CA, USA) and sequenced on an NovaSeq X Plus (Illumina, San Diego, CA, USA) at the Next-Generation Sequencing core facility, the Research Institute for Microbial Diseases of Osaka University. Alignment to the GRCh37 human reference genome, variant calling and annotation were performed using the Franklin platform (Genoox, Tel Aviv, Israel). Utilizing population databases including the Genome Aggregation Database (gnomAD v2.1.1), and the Japanese Multi Omics Reference Panel (jMorp; 60KJPN), we focused on rare variants with a minor allele frequency (MAF) < 0.01. All rare non-synonymous, nonsense, insertion/deletion, or splice-site variants were evaluated through whole-exome sequencing. This variant (NM_013254.4:c.993-2A>C) was analyzed by SpliceAI (https://spliceailookup.broadinstitute.org/), suggesting that it may produce splice abnormalities due to exon 9 skipping and intron 8 retention (Accepter Loss: 0.82, Donor Loss: 0.12, Accepter Gain: 0.52). For variant evaluation, reverse transcription-polymerase chain reaction (RT-PCR) was performed using RNA extracted from patient-derived lymphoblastoid cell lines (LCL) with RNeasy Plus Mini kit (QIAGEN, Valencia, CA, USA). The first strand cDNA was generated using the PrimeScript 1st strand cDNA Synthesis kit (Takara, Shiga, Japan) and was used as a template for RT-PCR. The primer sequences used for RT-PCR were as follows: Forward 5'-AGTGTTGGGGTTTTGACCAG-3', Reverse 5'-CCCCTGTTATTGCCTTAGCC-3'. As a result, an intron 8 retention transcript was observed that generates a new acceptor site in intron 8. This transcript is expected to produce an early termination codon based on the frameshift. The observation of aberrant transcripts dominantly in the cycloheximide-treated cells also suggested that their expression was reduced by a nonsense-mediated mRNA decay. Although SpliceAI predicted exon 9 skipping, this was not detected in our analysis using LCL cells. Given the current data, we cannot rule out the possibility of a tissue-specific transcript variant.

To test for the presence of a GGGGCC expansion in *C9orf72*, repeat-primed polymerase chain reaction (RP-PCR) was performed as previously described.^1^

This investigation was approved by the institutional ethics committee of Tokushima University and was conducted in accordance with the Declaration of Helsinki. Written informed consent was obtained from the patient, and patient anonymity was preserved.

**Reference**

1. DeJesus-Hernandez M, Mackenzie IR, Boeve BF, et al. Expanded GGGGCC hexanucleotide repeat in noncoding region of C9ORF72 causes chromosome 9p-linked FTD and ALS. *Neuron* 2011;72(2):245-256; doi:10.1016/j.neuron.2011.09.011.
